# Virulence and Genomic Feature of a Virulent *Klebsiella pneumoniae* Sequence Type 14 Strain of Serotype K2 Harboring *bla*_NDM–5_ in China

**DOI:** 10.3389/fmicb.2017.00335

**Published:** 2017-03-23

**Authors:** Yan-fang Mei, Pan-pan Liu, La-Gen Wan, Yang Liu, Lian-hui Wang, Dan-dan Wei, Qiong Deng, Xian-wei Cao

**Affiliations:** ^1^Department of Clinical Microbiology, First Affiliated Hospital of Nanchang University, Nanchang University,Nanchang, China; ^2^Department of Infection Control, First Affiliated Hospital of Nanchang University, Nanchang University,Nanchang, China

**Keywords:** carbapenemase, *K. pneumoniae*, metallo-β-lactamases, virulence factor, serotype

## Abstract

The objective of this study was to reveal the molecular mechanism involved in carbapenem resistance and virulence of a K2 *Klebsiella pneumoniae* clinical isolate 24835. The virulence of the strain was determined by *in vitro* and *in vivo* methods. The *de novo* whole-genome sequencing technology and molecular biology methods were used to analyze the genomic features associated with the carbapenem resistance and virulence of *K. pneumoniae* 24835. Strain 24835 was highly resistant to carbapenems and belonged to ST14, exhibited hypermucoviscous and unique K2-aerobactin-kfu-rmpA positive phenotype. As the only carbapenemase gene in strain 24835, *bla*_NDM–5_ was located on a 46-kb IncX3 self-transmissible plasmid, which is a very close relation of pNDM-MGR194 from India. Genetic context of *bla*_NDM–5_ in strain 24835 was closely related to those on IncX3 plasmids in various Enterobacteriaceae species in China. The combination of multiple virulence genes may work together to confer the relative higher virulence in *K. pneumoniae* 24835. Significantly increased resistance to serum killing and mice mortality were found in the virulent New Delhi metallo-β-lactamase (NDM)-producing *K. pneumoniae* strain compared to the other NDM-producing *K. pneumoniae* strain. Our study provides basic information of phenotypic and genomic features of *K. pneumoniae* 24835, a strain displaying carbapenem resistance and relatively high level of virulence. These findings are concerning for the potential of NDM-like genes to disseminate among virulent *K. pneumoniae* isolates.

## Introduction

*Klebsiella pneumoniae* is one of the most frequent causes of both health care- and community-associated infections, including pyogenic liver abscess, urinary tract infections, bacteremia, and pneumonia (Keynan and Rubinstein, 2007). One changing trend is the increase of antimicrobial resistance in hypervirulent *K. pneumoniae*, especially the emergence of carbapenemase producing hypervirulent *K. pneumoniae* (CP-hvKP), which has increasingly caused serious global public health concern (Zhang et al., 2015).

New Delhi metallo-β-lactamase (NDM) carbapenemase is increasingly being reported in different parts of the world (Nordmann et al., 2011). A few of them have been tested for their enzymatic kinetics, which denotes that amino acid substitution is a major source of metallo-β-lactamase (MBL) activity extension (Dortet et al., 2014). NDM-5 differs from existing enzymes due to substitutions at positions 88 (Val→Leu) and 154 (Met→Leu), which appear to confer increased resistance to carbapenems and broad-spectrum cephalosporins. After the first identification in China (Yang et al., 2014), the incidence of NDM-5 expression by *Escherichia coli* has increased dramatically. However, NDM-5 carbapenemases remain very rare in *K. pneumoniae*. Moreover, little is known about the virulence potential of NDM-5-producing *K. pneumoniae*.

Although genomes of many human-source hvKP (e.g., NTUH K2044, KPNIH31, Kp13, 65BO, and KCTC 2242) have been sequenced, only three genomes of K2-hvKP (KP617, PittNDM01, and CG43) have been obtained (Wu et al., 2009; Ramos et al., 2014; Struve et al., 2015). The genome sequences of K2-hvKP isolates with multidrug resistance and high virulence have not been published previously. In order to investigate the mechanisms involved in the multidrug resistance and increased virulence in K2-hvKP in China, the genomic profile of a K2-hvKP isolate with relative higher virulence and carbapenem resistance was determined by the *de novo* sequencing technology in the present study.

## Materials and Methods

### Patient and Isolates

In April 4, 2015, a 65-year-old female was hospitalized in the First Affiliated Hospital of Nanchang University, China, for a cerebral infarction. In addition, the patient has the following diseases: coronary atherosclerotic heart disease, hypertension, type 2 diabetes, and hyperlipoidemia. At that time, the white blood cell (WBC) count was 8.2 × 10^9^/L with 65.1% granulocytes and 19.8% lymphocytes. However, on April 12, her WBC count increased to 15.5 × 10^9^/L with unusual 87.5% granulocytes and 6.3% lymphocytes, accompanied by a high temperature, breathing difficulty, and brick red foamy blood sputum, and followed by respiratory distress syndrome. Furthermore, the patient developed pneumonia and bacteriuria that was empirically treated with ceftazidime, ciprofloxacin, and metronidazole. The clinical status of the patient worsened necessitating intubation and mechanical ventilation on day 8, and her transfer on day 11 to the intensive care unit and died at 4 am, on April 16.

*Klebsiella pneumoniae* strain 24835 was isolated from midstream urine specimen. The isolate was identified using the VITEK 2 (BioMérieux, France). Further identification of *K. pneumoniae* was performed by partially sequencing the 16S rRNA gene (Lane, 1991). In addition, the hypermucoviscous (HV) phenotype was tested by evaluating the formation of a viscous string (positive test, >0.5 cm in length) that was stretched using an inoculation loop (Shon et al., 2013).

### Antimicrobial Susceptibility Testing and Detection of Resistance Mechanisms

Antimicrobial susceptibilities for the *bla*_NDM–5_ positive isolates and transconjugants were initially tested using the VITEK 2 system (BioMérieux, France) and then were followed by measuring minimum inhibitory concentration (MIC) using the microbroth dilution method (for ampicillin/sulbactam, piperacillin/tazobactam, cefazolin, cefotetan, ceftazidime, cefepime, imipenem, ertapenem, ciprofloxacin, levofloxacin, gentamicin, amikacin, and aztreonam) and E-test (AB bioMérieux, Sweden) (for tigecycline), respectively. The standard microbroth methods were performed according to the guideline M07-A9 of the Clinical and Laboratory Standards Institute [CLSI] (2015). E-tests were conducted according to packet insert instructions using Mueller–Hinton agar (MHA). Fresh bacterial colonies taken directly from MHA plates that were incubated at 37°C for 16–20 h were re-suspended in sterile Mueller–Hinton broth to obtain a suspension of 0.5 McFarland turbidity. *E. coli* ATCC 25922 was used as the quality control. The MIC results were interpreted according to the CLSI guidelines M100-S25 (Clinical and Laboratory Standards Institute [CLSI], 2015). The Food and Drug Administration (FDA) breakpoint was used for tigecycline.

Double disc synergy tests (DDSTs) using imipenem/ethylenediaminetetraacetic acid (EDTA) and aztreonam and amoxicillin/clavulanic acid were employed to screen for metallo-β-lactamases and extended spectrum beta-lactamases (ESBLs), respectively. Isolate was screened by polymerase chain reaction (PCR) amplification using a panel of primers for the detection of MBLs, *Klebsiella pneumoniae* carbapenemases (KPCs), OXA-48, ESBLs, including the SHV, TEM, CTX-M, and IBC/GES enzymes, and plasmid-mediated AmpCs (D’Andrea et al., 2006; Queenan and Bush, 2007; Andrade et al., 2010).

### *De novo* Whole-Genome Sequencing of *K. pneumoniae* 24835

Total genomic DNA of *K. pneumoniae* 24835 was extracted using QIAamp DNA Mini Kit (Qiagen, Hilden, Germany) according to the manufacturer’s protocol. The genomic DNA was sent to Shanghai Biotechnology Corporation for the *de novo* whole-genome sequencing. A combination of Illumina MiSeq (300 bp paired-end, 12 million reads) and PacBio (10 kb fragment library, 356,001 reads) sequencing data were used to assemble the genome using SPAdes version 3.1.0 (Nurk et al., 2013). The Prokka program was employed for annotating the genomic sequence. Gene prediction was carried out using Glimmer 3.02, tRNA prediction with tRNAscan-SE, and rRNA prediction with HMMER, while basic local alignment search tool (BLAST) searches were performed against several databases including CatFam, COG, NCBI RefSeq, and SEED.

The acquired antimicrobial resistance genes were identified by uploading assembled genomes to the Resfinder server v2.1^1^. The other genes relating to resistance and virulence were detected using the mapping unit of CLC Genomics Workbench to map reads and/or by blasting assembled genomes to a pseudomolecule generated by concatenating a set of *K. pneumoniae* genes. Scaffolds with resistance-related and virulence genes were blasted against GenBank to identify their genetic location. The sequence data from *K. pneumoniae* 24835 was compared to the results of antimicrobial resistance and virulence to identify genetic factors that correspond to the observed mechanisms.

### Plasmid Analysis and Horizontal Gene Transfer of pNDM-5

Horizontal gene transfer of pNDM-5 was evaluated by transconjugation assays using 10^6^ colony forming units (cfu) of 24835 as a donor and 10^6^ cfu of *E. coli* J53 as recipients. After 24 h incubation, transconjugants were selected on MHA containing sodium azide (100 μg/ml) and imipenem (1 μg/ml). The presence of *bla*_NDM–5_ in transconjugants was confirmed using PCR and enterobacterial repetitive intergenic consensus polymerase chain reaction (ERIC-PCR) was used for further distinguishing transconjugants from the donor strain. Plasmid DNA that was prepared from the transconjugant using alkaline lysis was subjected to PCR-based replicon typing (Carattoli et al., 2005; Johnson et al., 2012). The resultant plasmids were annotated using the Prokaryotic 6 Genomes Automatic Annotation Pipeline (PGAAP) available at NCBI^2^. The plasmid was compared to publicly available plasmid references using BLAST at GenBank^3^. The plasmid comparison and visualization were generated by Easyfig (version 2.2.2) (Sullivan et al., 2011).

### Pulsed Field Gel Electrophoresis (S1-PFGE)

Pulsed field gel electrophoresis (S1-PFGE) was performed to determine the number and size of plasmids carried by strain 24835 as described previously. Briefly, agarose plugs containing whole-cell DNA of strain 24835 were treated with 8 U of S1 nuclease (Fermentas, Thermo Scientific, Waltham, MA, USA) and the reaction was stopped by adding 0.5 M EDTA (pH = 8). PFGE was conducted with a 1% SeaKem Gold agarose gel (Lonza, Basal, Switzerland) using a CHEF DRII system (Bio-Rad, Hercules, CA, USA) at 14°C, with a 6-V/cm current and run times of 12 h at switch time of 5–40 s followed by 8 h at switch time of 3–8 s. MidRange I PFG Marker (NEB, Ipswich, MA, USA) was used for size estimation.

### Multilocus Sequence Typing

Multilocus sequence typing (MLST) on the *K. pneumoniae* were performed as previously described (Liu et al., 2015).

### Serum Killing Assay

Human blood was obtained from 10 healthy individuals. Pooled serum was separated and stored in small volumes at −80°C until use. Serum killing assay was performed as previously described. An inoculums of 25 μL (adjusted to 10^6^ cfu/ml) prepared from the mid-log phase was diluted by 0.9% saline, and was added to 75 μL of pooled human sera contained in a 10 × 75 mm Falcon polypropylene tube (BD Biosciences, Franklin Lakes, NJ, USA). Serial dilutions were plated on MHA for 0, 1, 2, and 3 h to obtain colony counts.

♦ Grade 1 is viable counts <10% of the inoculum after 1 and 2 h, and <0.1% after 3 h.
♦ Grade 2 is viable counts 10–100% of the inoculum after 1 h and <10% after 3 h.
♦ Grade 3 is viable counts that exceeded those of the inoculum after 1 h, but <100% after 2 and 3 h.
♦ Grade 4 is viable counts >100% of the inoculum after both 1 and 2 h, but <100% after 3 h.
♦ Grade 5 is viable counts >100% of the inoculums 1, 2, and 3 h, but that decreased during the third hour.
♦ Grade 6 is viable counts that exceeded those of the inoculum after 1, 2, and 3 h, and increased throughout this time period.

Each strain was tested at least three times, and the mean results were expressed as percent inoculums. The results were expressed as percentage of inoculation and the responses in terms of viable counts were graded from 1 to 6, as previously described (Abate et al., 2012). A strain was defined as serum sensitive at grades of 1–2, intermediately sensitive at grades of 3–4, and resistant at grades of 5–6. *K. pneumoniae* ATCC 700603 and 19432 (NDM-1 positive) strains are used for comparison in the serum killing assay.

### Mouse Lethality Assay

To determine the 50% lethal dose (LD50), pathogen-free, 6- to 8-week-old, male BALB/c mice were obtained from Nanchang University Animal Center. Six mice were used as a sample population for each bacterial concentration. Ten-fold serial dilution of cfu of *K. pneumoniae* was made from a starting concentration of 10^6^ cfu/ml -10 cfu/ml, and BALB/c mice were infected intraperitoneally with 0.1 ml of each concentration (Zhang et al., 2015). Symptoms and mortality rates were observed for 14 days. The exact inoculation dose was confirmed on Luria-Bertani (LB) agar and the LD50 was calculated as described by Reed and Muench (1938). Survival curves were assessed by Kaplan–Meier analysis and log-rank test. *P* < 0.05 was considered statistically significant. *K. pneumoniae* ATCC 700603 and 19432 (NDM-1 positive) strains are used for comparison in the mouse lethality assay.

### Nucleotide Sequence Accession Numbers

The whole-genome shotgun project of *K. pneumoniae* 24835 has been deposited at GenBank under the accession number CP014004. The annotated sequences of p24835-NDM5 and p24835-CTXM15 have been submitted to GenBank nucleotide sequence database under accession numbers CP014005 and CP014006, respectively.

## Results

### Isolation of a NDM-5 Positive *K. pneumoniae* Isolate

**FIGURE 1:**
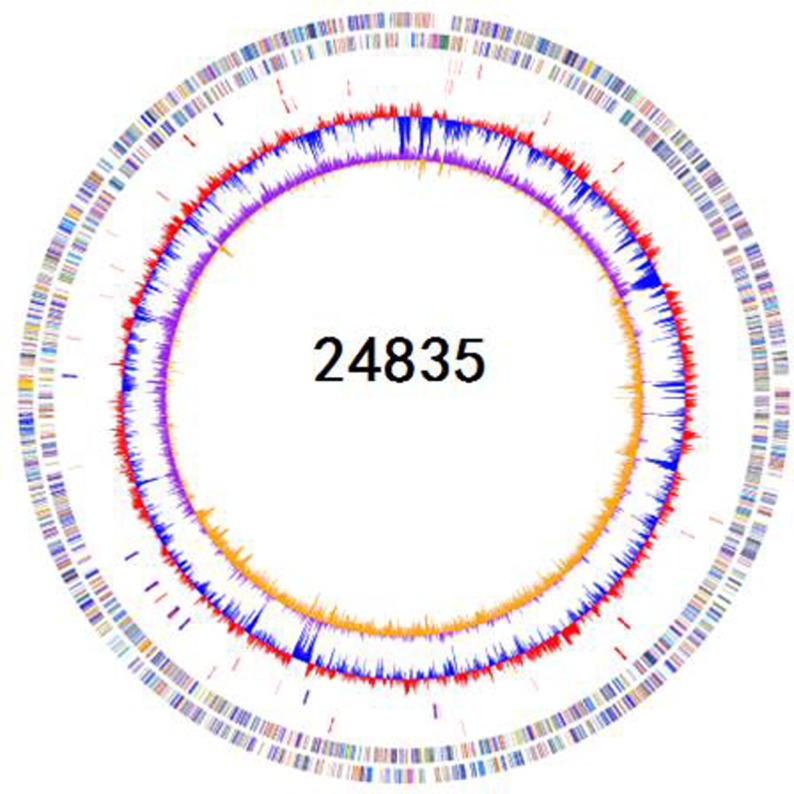
**Schematic circular genome of *K. pneumoniae* 24835 strain.** From outside to inside, there were four rings. The first circle shows COG functional annotations. The second circle are rRNA and tRNA (red-tRNA, blue-rRNA). The third circle is the GC contents. The fourth circle is the GC skew with + value (orange) and -value (purple).

A carbapenem-resistant *K. pneumoniae* isolate (termed 24835) at >10^6^ cfu/ml was recovered from urine on April 13, 2015. The patient was isolated in a single room and treated according to national and local infection control guidelines. This included an extensive terminal disinfection of the sickroom after discharge of the patient. The patient had no recent history of travel outside. 24835 were resistant to all antibiotics recommended by CLSI for the susceptibility testing of *Enterobacteriaceae*, with both isolates exhibiting identical MIC values (**Figure 1** and Supplementary Table S2). Phenotypic ESBL and AmpC screening by DDST was positive, and PCR analyses and subsequent sequencing revealed the presence of *bla*_TEM–1B_, *bla*_SHV–28_, and *bla*_CTX–M–15_. The DDST with imipenem/EDTA was positive, suggesting the production of a metallo-β-lactamase, which was verified by PCR detection of *bla*_NDM–5_.

### Genome Sequencing and Analysis

A total of 4,001,934 (1,208,584,068 bp) paired-end reads were generated using Illumina-HiSeq 2500. Using the PacBio RS II platform, 64,416 (941,657,914 bp) raw reads were produced. The complete genome of 24835 consists of a 5,387,996-bp circular chromosome and two plasmids of 101,030 and 46,161 bp in size. The genomic features of 24835 and the reference strains are summarized in **Figure 1**. Based on a Prokka analysis, 5191 putative open reading frames (ORFs) and 84 RNA genes on the circular chromosome (**Figure 1**; Supplementary Table S1), 108 putative ORFs on plasmid 1, and 49 putative ORFs on plasmid 2 were identified.

Nineteen antibiotic resistance genes were identified in the genome of 24385 strain. The β-lactam resistance genes in the 24385 genome were *bla*_TEM–1B_ and *bla*_SHV–28_ in the chromosome, *bla*_CTX–M–15_ in plasmid 1, and *bla*_NDM–5_ in plasmid 2 (Supplementary Figure S1). The clinical features of *K. pneumoniae* infections depend on the virulence factors expressed by the infecting strain (Yu et al., 2007). A BLAST search was performed against virulence factors database (VFDB) to identify 120 virulence factors harbored by the 24385 strain. The 120 virulence genes of 24385 strain were classified into 31 the following categories: Iron uptake (32 genes), Immune evasion (12 genes), Endotoxin (11 genes), Adherence (11 genes), Fimbrial adherence determinants (8 genes), Toxin (7 genes), Antiphagocytosis (6 genes), Regulation (5 genes), Acid resistance (3 genes), Anaerobic respiration (2 genes), Cell surface components (2 genes), and Secretion system (2 genes). Among the 120 virulence genes identified, 8 genes were lipopolysaccharide (LPS)-related genes and 4 genes were capsular polysaccharide-related.

### Identification, Transferability, and Genetic Context of *bla*_NDM–5_ in 24835

*bla*_NDM–5_ was successfully transferred to *E. coli* J53, suggesting its localization on a plasmid. Strikingly, the conjugation frequency was high. Plasmid p24835-NDM5 was readily transferred from *K. pneumoniae* 24835 to *E. coli* J53Az^R^ with a frequency of (2.4 ± 0.9) × 10^-4^ per donor cell at 37°C.

**FIGURE 2:**
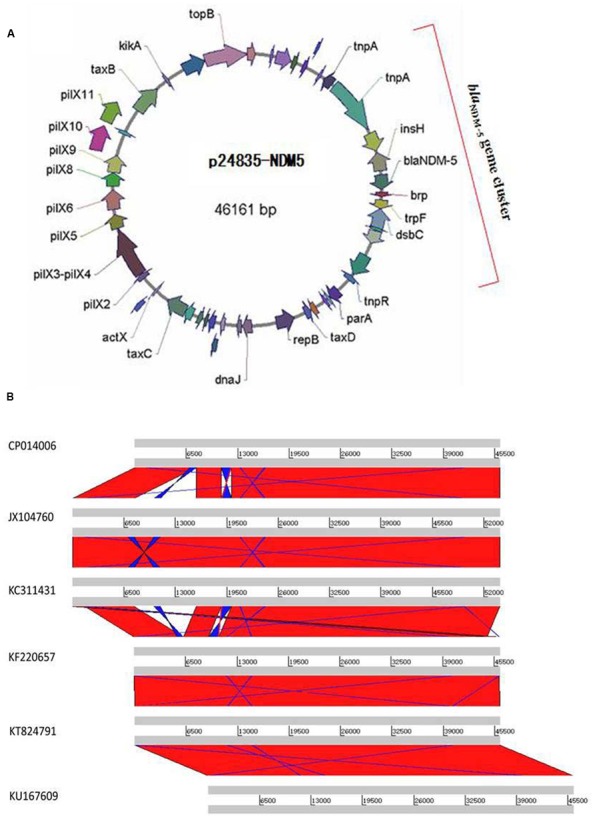
**Plasmid analysis of p24835-NDM5. (A)** Schematic map of plasmid p24835-NDM5, ORFs are color coded according to predicted function as indicated in the associated key. Arrows show the direction of transcription; **(B)** comparative analysis of plasmid structures between p524835-NDM (CP014006) and the reference plasmids pNDM-MGR194 (KF220657), pNDM-QD29 (KU167609), pKN5047 (KC311431), pNDM-HN380 (JX104760), and pEc1929 (KT824791). The identical regions are highlighted in red.

Transconjugants expressing NDM-5 were non-susceptible to all tested β-lactam antibiotics, including aztreonam, suggesting the presence of other β-lactamases besides NDM-5 on the plasmid (Supplementary Table S2). Transconjugants were positive for *bla*_CTX–M–15_, but negative for *bla*_SHV–28_, confirming the results of phenotypic testing. NDM-carrying transconjugants were furthermore sensitive to gentamicin and amikacin, indicating the absence of aminoglycoside resistance genes. Plasmid p24835-NDM5 carrying the *bla*_NDM–5_ gene was 46,161 bp in length, with an average GC content of 52.04% (**Figure 2A**). It possessed 114 ORFs, including those corresponding to the replication, conjugation, and antibiotic resistance modules. Its scaffold corresponded to that of IncX3-type plasmids, commonly identified among enterobacterial isolates. In particular, it showed similarities with plasmid pNDM-MGR194, which was recently identified at the origin of the *bla*_NDM–1_ gene acquisition in a *K. pneumoniae* isolate in India (Krishnaraju et al., 2015). Comparative analysis of the genetic contexts of *bla*_NDM–5_ in this IncX3 plasmid and the previously reported plasmids revealed some differences (**Figure 2B**).

### Detection of Virulence-Associated Determinants, Serum Killing, and Mouse Lethality Assay

MLST showed that 24835 isolate was ST14. The strain was confirmed as serotype K2 and aerobactin-kfu-rmpA positive (Supplementary Figure S2). The identified virulence determinants may have contributed to the infection and or colonization of *K. pneumoniae* 24835 in the urinary tract. Furthermore, the isolate grew as positive HV phenotype with a mucoviscous string >5 mm in length from the colony on the blood agar plate (Supplementary Figure S2), however, the fact was observed after initial isolation of the bacterium, and, thereafter, the characteristic decreased.

**FIGURE 3:**
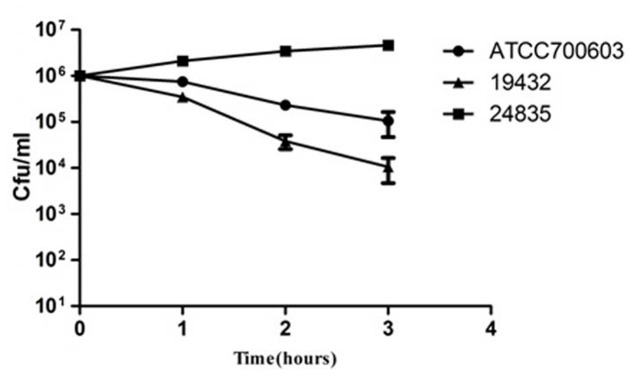
**Serum killing assay of 24835, 19432, and ATCC 700603 strains.** Survival of each strain was assessed by enumerating viable counts at 0, 1, 2, and 3 h of incubation in the pooled human sera at 37°C. There was a significant increase in the growth of the strain 24835. Data shown are mean ± SEM of triplicates.

**FIGURE 4:**
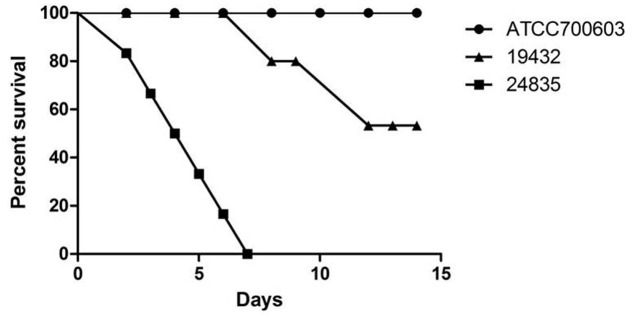
**Mouse lethality assay of 24835, 19432, and ATCC 700603 strains.** The mortality of mice after intraperitoneal injection of all strains was observed over 14 days. Data points represented the percentage of mice survival in each group (*n* = 6 mice per strain). A significant difference in mice lethality was identified between strain 24835 and the other strains (*P* < 0.001).

Serum killing resistance (grade 6) was found in strain 24835. The other two isolates (ATCC 700603 and 19432) showed serum sensitive (grade 1 or 2) (**Figure 3**). Mouse lethality assay was used to study the virulence *in vivo*. The results showed that LD50 of >10^6^ cfu, which indicates low virulence, was identified in a NDM-1-producing strain 19432 and ATCC 700603. In contrast, strain 24835 was shown hypervirulent, as all mice injected with this strain died within 7 days at a low concentration (**Figure 4**). LD50 of strain 24835 was 105.2 cfu (<10^3^ cfu), exhibiting a high virulence level of “+++” according to previously described, which is in line with studies from Wei et al. (2016) who also reported an LD50, 120 cfu for highly virulent KPC-producing strain. This shows that the NDM-5-containing clinical isolate 24835 was more virulent than ATCC 700603 and the other, NDM-1 positive, *K. pneumoniae* strain 19432.

## Discussion

So far, compared to serotype K1 HvKP, not much has been studies about K2 strains. Serotype K2 hvKP (K2-hvKP) also has significant clinical importance due to their ability to cause life threatening and metastatic infections (Zhang et al., 2015). Clonal complexes of K2-hvKP and multidrug resistant strains had been considered independent until 2015, when carbapenem-resistant K2-hvKP were first identified in China (Zhang et al., 2015). The carbapenem resistant K2-hvKP may greatly threaten human public health, therefore it is important to investigate the virulence potential and mechanism involved in carbapenem resistance and virulence of K2-hvKP.

24835 had multidrug resistance to fluoroquinolones, carbapenem, cephalosporins, monobactams, and fosfomycin. The *oqxA* and *oqxB* gene contributed to its high-level resistance to fluoroquinolones (Bialek-Davenet et al., 2015). High level cephalosporins and monobactams resistance in *K. pneumoniae* 24835 was likely due to *bla*_CTX–M–15_, *bla*_SHV–28_, and *bla*_TEM–1B_ (Nielsen et al., 2011; Calbo and Garau, 2015; Tokajian et al., 2015). The *fosA3* gene contributed to its high-level resistance to fosfomycin (Jiang et al., 2015). The high-level resistance to carbapenem was mediated by *bla*_NDM–5_ gene which was located in p24835-NDM5 plasmid (Sun et al., 2016). The p24835-NDM5 plasmid in 24835 was identical to the one found in pNDM-MGR194 (Shin et al., 2016). As a mobile gene element, p24835-NDM5 plasmid may act as a vehicle to pick up and spread multiple antibiotic resistance genes and virulence genes in *K. pneumoniae* (Burmølle et al., 2012). The *aadA2* gene in 24835 could explain its intermediate to tobramycin.

Generally, it has been through that multidrug resistance is often associated with higher fitness costs or less virulence (Hennequin and Robin, 2016). However, our study suggested that the multidrug resistant 24835 had higher virulence than the reference strain ATCC 700603 and 19432 that are not multidrug resistant. Previous studies indicated that an association of CTX-M enzyme production with virulence and fitness in *K. pneumoniae* (Ranjbar et al., 2016). A recent study further revealed that the expression of both efflux systems and porins is a key factor not only for antibiotic resistance but also virulence potential in *K. pneumoniae* (Lin et al., 2016). However, another study suggests that known virulence factors such as K1, K2, and K5 capsular polysaccharides, rmpA and the aerobactin gene were absent in KPC-producing isolates and these strains present low virulence in a murine lethality model (Chiang et al., 2016). Some of the clinical investigation data indicated the positive correlation between virulence and resistance, but others indicated the negative correlation (Liu et al., 2013; McLaughlin et al., 2014). Our study revealed the relationship between multidrug resistance and virulence of *K. pneumoniae* 24835, indicating that complex mechanisms may work together to cause multidrug resistance and the relative higher virulence.

24835 contained some special virulence-associated genes and proteins, including *ureA*, *fimH*, *uge*, *wabG*, *mrkD*, Quorum sensing system, capsule polysaccharide protein, exoenzyme and iron uptake system. The *urea* is related to the urease operon, which is involved in urea metabolism and required for efficient bacterial gastrointestinal colonization. The *fimH*, encoding adhesin, may play an important role on the colonization, invasion, and biofilm formation of 24835 (Candan and Aksöz, 2015). The presence of *uge* may contribute to the expression of smooth LPS with O antigen molecules and capsule polysaccharide on the cell surface, yielding the ability to produce a UTI and virulence during sepsis and pneumonia of *K. pneumoniae* 24835. The *wabG* may play a critical role in the cell attachment of capsular polysaccharide and contribute to the biosynthesis of the core LPS and encapsulated cell (Andrade et al., 2014). The *mrkD* encodes the type 3 fimbriae adhesin, which facilitates adhesion to the basement membranes of several human tissue (Brisse et al., 2009). The quorum sensing system, capsule polysaccharide protein, exoenzyme and iron uptake system may enhance the invasion, colonization, and biofilm formation of 24835 in reservoir hosts and mediate the virulence of 24835 by evading host immune response. These results also show the emergence of dual-risk *K. pneumoniae* strain combining both virulence and multidrug resistance features. Therefore, acquisition of an important mechanism of carbapenem resistance, such as NDM-5 in the virulent strain, may be a concern in the future. Future experiments are needed to find the deeper molecular mechanisms and confirm the function of some important gene elements on multidrug resistance and relative higher virulence.

## Ethics Statement

The experimental protocols were approved by the Animal Care and Protection Committee of Nanchang University.

## Author Contributions

YL, YFM, PPL, LGW, DDW, LHW, QD, and XWC performed the laboratory measurements. YL and LGW made substantial contributions to conception and design. XWC and YL revised the manuscript critically for important intellectual content. YFM, PPL, LHW, QD, and DDW participated in experimental design and data analysis. YL drafted the manuscript. All authors read and approved the final manuscript.

## Conflict of Interest Statement

The authors declare that the research was conducted in the absence of any commercial or financial relationships that could be construed as a potential conflict of interest. The reviewer YS and handling Editor declared their shared affiliation, and the handling Editor states that the process nevertheless met the standards of a fair and objective review.

## Footnotes

1 http://cge.cbs.dtu.dk/services/ResFinder-2.1/

2 http://www.ncbi.nlm.nih.gov/

3 www.ncbi.nlm.nih.gov/genbank/

## References

[B1] Abate G. Koh T. H. Gardner M. Siu L. K. (2012). Clinical and bacteriological characteristics of *Klebsiella pneumoniae* causing liver abscess with less frequently observed multi-locus sequences type, ST163, from Singapore and Missouri, US. *J. Microbiol. Immunol. Infect.* 45 31–36. 10.1016/j.jmii.2011.09.002 22138655

[B2] Andrade L. N. Minarini L. A. Pitondo-Silva A. Climaco E. C. Palazzo I. C. Medeiros M. I. (2010). Determinants of beta-lactam resistance in meningitis-causing *Enterobacteriaceae* in Brazil. *Can. J. Microbiol.* 56 399–407. 10.1139/W10-020 20555402

[B3] Andrade L. N. Vitali L. Gaspar G. G. Bellissimo-Rodrigues F. Martinez R. Darini A. L. (2014). Expansion and evolution of a virulent, extensively drug-resistant (polymyxin B-resistant), QnrS1-, CTX-M-2-, and KPC-2-producing *Klebsiella pneumoniae* ST11 international high-risk clone. *J. Clin. Microbiol.* 52 2530–2535. 10.1128/JCM.00088-14 24808234 PMC4097695

[B4] Bialek-Davenet S. Lavigne J. P. Guyot K. Mayer N. Tournebize R. Brisse S. (2015). Differential contribution of AcrAB and OqxAB efflux pumps to multidrug resistance and virulence in *Klebsiella pneumoniae*. *J. Antimicrob. Chemother.* 70 81–88. 10.1093/jac/dku340 25193085

[B5] Brisse S. Fevre C. Passet V. Issenhuth-Jeanjean S. Tournebize R. Diancourt L. (2009). Virulent clones of *Klebsiella pneumoniae*: identification and evolutionary scenario based on genomic and phenotypic characterization. *PLoS ONE* 4:e4982 10.1371/journal.pone.0004982 PMC2656620 19319196

[B6] Burmølle M. Norman A. Sørensen S. J. Hansen L. H. (2012). Sequencing of IncX-plasmids suggests ubiquity of mobile forms of a biofilm-promoting gene cassette recruited from *Klebsiella pneumoniae*. *PLoS ONE* 7:e41259 10.1371/journal.pone.0041259 PMC3402527 22844447

[B7] Calbo E. Garau J. (2015). The changing epidemiology of hospital outbreaks due to ESBL-producing *Klebsiella pneumoniae*: the CTX-M-15 type consolidation. *Future Microbiol.* 10 1063–1075. 10.2217/fmb.15.22 26059626

[B8] Candan E. D. Aksöz N. (2015). *Klebsiella pneumoniae*: characteristics of carbapenem resistance and virulence factors. *Acta Biochim. Pol.* 62 867–874. 10.18388/abp.2015_1148 26637376

[B9] Carattoli A. Bertini A. Villa L. Falbo V. Hopkins K. L. Threlfall E. J. (2005). Identification of plasmids by PCR-based replicon typing. *J. Microbiol. Methods* 63 219–228. 10.1016/j.mimet.2005.03.018 15935499

[B10] Chiang T. T. Yang Y. S. Yeh K. M. Chiu S. K. Wang N. C. Lin T. Y. (2016). Quantification and comparison of virulence and characteristics of different variants of carbapenemase-producing *Klebsiella pneumoniae* clinical isolates from Taiwan and the United States. *J. Microbiol. Immunol. Infect.* 49 83–90. 10.1016/j.jmii.2015.08.011 26514941

[B11] Clinical and Laboratory Standards Institute [CLSI] (2015). *Performance Standards for Antimicrobial Susceptibility Testing; Twenty-Third Informational Supplement. M100-S24* Wayne, PA: Clinical and Laboratory Standards Institute.

[B12] D’Andrea M. M. Nucleo E. Luzzaro F. Giani T. Migliavacca R. Vailati F. (2006). CMY-16, a novel acquired AmpC-type beta-lactamase of the CMY/LAT lineage in multifocal monophyletic isolates of *Proteus mirabilis* from northern Italy. *Antimicrob. Agents Chemother.* 50 618–624. 10.1128/AAC.50.2.618-624.2006 16436718 PMC1366893

[B13] Dortet L. Poirel L. Nordmann P. (2014). Worldwide dissemination of the NDM-type carbapenemases in gram-negative bacteria. *Biomed. Res. Int.* 2014:249856 10.1155/2014/249856 PMC3984790 24790993

[B14] Hennequin C. Robin F. (2016). Correlation between antimicrobial resistance and virulence in *Klebsiella pneumoniae*. *Eur. J. Clin. Microbiol. Infect. Dis.* 35 333–341. 10.1007/s10096-015-2559-7 26718943

[B15] Jiang Y. Shen P. Wei Z. Liu L. He F. Shi K. (2015). Dissemination of a clone carrying a fosA3-harbouring plasmid mediates high fosfomycin resistance rate of KPC-producing *Klebsiella pneumoniae* in China. *Int. J. Antimicrob. Agents* 45 66–70. 10.1016/j.ijantimicag.2014.08.010 25450805

[B16] Johnson T. J. Bielak E. M. Fortini D. Hansen L. H. Hasman H. Debroy C. (2012). Expansion of the IncX plasmid family for improved identification and typing of novel plasmids in drugresistant *Enterobacteriaceae*. *Plasmid* 68 43–50. 10.1016/j.plasmid.2012.03.001 22470007

[B17] Keynan Y. Rubinstein E. (2007). The changing face of *Klebsiella pneumoniae* infections in the community. *Int. J. Antimicrob. Agents* 30 385–389. 10.1016/j.ijantimicag.2007.06.019 17716872

[B18] Krishnaraju M. Kamatchi C. Jha A. K. Devasena N. Vennila R. Sumathi G. (2015). Complete sequencing of an IncX3 plasmid carrying blaNDM-5 allele reveals an early stage in the dissemination of the blaNDM gene. *Indian J. Med. Microbiol.* 33 30–38. 10.4103/0255-0857.148373 25559999

[B19] Lane D. J. (1991). “16S/23S rRNA sequencing,” in *Nucleic Acid Techniques in Bacterial Systematics*, eds Stackebrant E. Goodfellow M. (New York, NY: John Wiley & Sons), 115–175.

[B20] Lin Y. T. Huang Y. W. Huang H. H. Yang T. C. Wang F. D. Fung C. P. (2016). In vivo evolution of tigecycline-non-susceptible *Klebsiella pneumoniae* strains in patients: relationship between virulence and resistance. *Int. J. Antimicrob. Agents* 48 485–491. 10.1016/j.ijantimicag.2016.07.008 27575728

[B21] Liu Y. Li X. Y. Wan L. G. Jiang W. Y. Yang J. H. Li F. Q. (2013). Virulence and transferability of resistance determinants in a novel *Klebsiella pneumoniae* sequence type 1137 in China. *Microb. Drug Resist.* 20 150–155. 10.1089/mdr.2013.0107 24236613

[B22] Liu Y. Wan L. G. Deng Q. Cao X. W. Yu Y. Xu Q. F. (2015). First description of NDM-1-, KPC-2-, VIM-2- and IMP-4-producing *Klebsiella pneumoniae* strains in a single Chinese teaching hospital. *Epidemiol. Infect.* 143 376–384. 10.1017/S0950268814000995 24762211 PMC9206769

[B23] McLaughlin M. M. Advincula M. R. Malczynski M. Barajas G. Qi C. Scheetz M. H. (2014). uantifying the clinical virulence of *Klebsiella pneumoniae* producing carbapenemase *Klebsiella pneumoniae* with a *Galleria mellonella* model and a pilot study to translate to patient outcomes. *BMC Infect. Dis.* 14:31 10.1186/1471-2334-14-31 PMC3897888 24428847

[B24] Nielsen J. B. Skov M. N. Jørgensen R. L. Heltberg O. Hansen D. S. Schønning K. (2011). Identification of CTX-M-15, SHV-28-producing *Klebsiella pneumoniae* ST15 as an epidemic clone in the Copenhagen area using a semi-automated Rep-PCR typing assay. *Eur. J. Clin. Microbiol. Infect. Dis.* 30 773–778. 10.1007/s10096-011-1153-x 21253799

[B25] Nordmann P. Poirel L. Walsh T. R. Livermore D. M. (2011). The emerging NDM carbapenemases. *Trends Microbiol.* 19 588–595. 10.1016/j.tim.2011.09.005 22078325

[B26] Nurk S. Bankevich A. Antipov D. Gurevich A. Korobeynikov A. Lapidus A. (2013). “Assembling genomes and mini-metagenomes from highly chimeric reads,” in *Research in Computational Molecular Biology*, eds Deng M. Jiang R. Sun F. Zhang X. (Berlin: Springer), 158–170.

[B27] Queenan A. M. Bush K. (2007). Carbapenemases: the versatile beta-lactamases. *Clin. Microbiol. Rev.* 20 440–458. 10.1128/CMR.00001-07 17630334 PMC1932750

[B28] Ramos P. I. Picão R. C. Almeida L. G. Lima N. C. Girardello R. Vivan A. C. (2014). Comparative analysis of the complete genome of KPC-2-producing *Klebsiella pneumoniae* Kp13 reveals remarkable genome plasticity and a wide repertoire of virulence and resistance mechanisms. *BMC Genomics* 15:54 10.1186/1471-2164-15-54 PMC3904158 24450656

[B29] Ranjbar R. Memariani H. Sorouri R. Memariani M. (2016). Distribution of virulence genes and genotyping of CTX-M-15-producing *Klebsiella pneumoniae* isolated from patients with community-acquired urinary tract infection (CA-UTI). *Microb. Pathog.* 100 244–249. 10.1016/j.micpath.2016.10.002 27725280

[B41] Reed L. J. Muench H. (1938). A simple method of estimating fifty per cent endpoints. *Am. J. Epidemiol.* 27 493–497. 10.1093/oxfordjournals.aje.a118408

[B30] Shin J. Baek J. Y. Cho S. Y. Huh H. J. Lee N. Y. Song J. H. (2016). blaNDM-5-bearing IncFII-type plasmids of *Klebsiella pneumoniae* sequence type 147 transmitted by cross-border transfer of a patient. *Antimicrob. Agents Chemother.* 60 1932–1934. 10.1128/AAC.02722-15 26824953 PMC4775946

[B31] Shon A. S. Bajwa R. P. Russo T. A. (2013). Hypervirulent (hypermucoviscous) *Klebsiella pneumoniae*: a new and dangerous breed. *Virulence* 4 107–118. 10.4161/viru.22718 23302790 PMC3654609

[B32] Struve C. Roe C. C. Stegger M. Stahlhut S. G. Hansen D. S. Engelthaler D. M. (2015). Mapping the evolution of hypervirulent *Klebsiella pneumoniae*. *mBio* 6 e00630-15. 10.1128/mBio.00630-15 PMC4513082 26199326

[B33] Sullivan M. J. Petty N. K. Beatson S. A. (2011). Easyfig: a genome comparison visualizer. *Bioinformatics* 27 1009–1010. 10.1093/bioinformatics/btr039 21278367 PMC3065679

[B34] Sun J. Yang R. S. Zhang Q. Feng Y. Fang L. X. Xia J. (2016). Co-transfer of blaNDM-5 and mcr-1 by an IncX3-X4 hybrid plasmid in *Escherichia coli*. *Nat. Microbiol.* 1 16176 10.1038/nmicrobiol.2016.176 27668643

[B35] Tokajian S. Eisen J. A. Jospin G. Farra A. Coil D. A. (2015). Whole genome sequencing of extended-spectrum β-lactamase producing *Klebsiella pneumoniae* isolated from a patient in Lebanon. *Front. Cell. Infect. Microbiol.* 5:32 10.3389/fcimb.2015.00032 PMC4389573 25905047

[B36] Wei D. D. Wan L. G. Deng Q. Liu Y. (2016). Emergence of KPC-producing *Klebsiella pneumoniae* hypervirulent clone of capsular serotype K1 that belongs to sequence type 11 in Mainland China. *Diagn. Microbiol. Infect. Dis.* 85 192–194. 10.1016/j.diagmicrobio.2015.03.012 27049969

[B37] Wu K. M. Li L. H. Yan J. J. Tsao N. Liao T. L. Tsai H. C. (2009). Genome sequencing and comparative analysis of *Klebsiella pneumoniae* NTUH-K2044, a strain causing liver abscess and meningitis. *J. Bacteriol.* 191 4492–4501. 10.1128/JB.00315-09 19447910 PMC2704730

[B38] Yang P. Xie Y. Feng P. Zong Z. Y. (2014). blaNDM-5 carried by an IncX3 plasmid in *Escherichia coli* sequence type 167. *Antimicrob. Agents Chemother.* 58 7548–7552. 10.1128/AAC.03911-14 25246393 PMC4249565

[B39] Yu V. L. Hansen D. S. Ko W. C. Sagnimeni A. Klugman K. P. von Gottberg A. (2007). Virulence characteristics of Klebsiella and clinical manifestations of *K. pneumoniae* bloodstream infections. *Emerg. Infect. Dis.* 13 986–993. 10.3201/eid1307.070187 18214169 PMC2878244

[B40] Zhang Y. W. Zeng J. Liu W. E. Zhao F. Hu Z. Zhao C. (2015). Emergence of a hypervirulent carbapenem-resistant *Klebsiella pneumoniae* isolate from clinical infections in China. *J. Infect.* 71 553–560. 10.1016/j.jinf.2015.07.010 26304687

