# Supplementary material for: Virulence and Genomic Feature of a Virulent Klebsiella pneumoniae Sequence Type 14 Strain of Serotype K2 Harboring blaNDM–5 in China
Source: Front Microbiol. 2017 Mar 23;8:335. doi: 10.3389/fmicb.2017.00335 (PMC5362587; doi:10.3389/fmicb.2017.00335)
Supplement: Supplementary file 1 [file Table_1.DOC]

**Table S1. Annotation of DNA sequences on plasmid pNDM5-24835**

| **position** | **start** | **end** | **strand** | **length** | **Function encoded** |
| --- | --- | --- | --- | --- | --- |
| p2 | 1 | 456 | + | 151 | MULTISPECIES: DNA-binding protein |
| p2 | 1281 | 1400 | + | 39 | MULTISPECIES: hypothetical protein |
| p2 | 1593 | 2414 | + | 273 | MULTISPECIES: hypothetical protein |
| p2 | 2524 | 2778 | + | 84 | MULTISPECIES: hypothetical protein |
| p2 | 3134 | 3346 | + | 70 | hypothetical protein |
| p2 | 3343 | 3489 | + | 48 | MULTISPECIES: hypothetical protein |
| p2 | 4121 | 4246 | + | 41 | no hits |
| p2 | 4998 | 4465 | - | 177 | transposase |
| p2 | 5154 | 8171 | + | 1005 | MULTISPECIES: transposase |
| p2 | 8391 | 9392 | + | 333 | hypothetical protein |
| p2 | 10481 | 9501 | - | 326 | MULTISPECIES: transposase |
| p2 | 10716 | 11528 | + | 270 | New Delhi metallo-beta-lactamase-5 |
| p2 | 11637 | 11897 | + | 86 | MULTISPECIES: bleMBL |
| p2 | 12070 | 12540 | + | 156 | MULTISPECIES: phosphoribosylanthranilate isomerase |
| p2 | 13582 | 12551 | - | 343 | MULTISPECIES: twin-arginine translocation (TAT) pathway signal sequence domain protein |
| p2 | 13721 | 13587 | - | 44 | hypothetical protein |
| p2 | 13780 | 14484 | + | 234 | MULTISPECIES: hypothetical protein |
| p2 | 15218 | 16438 | + | 406 | MULTISPECIES: transposase |
| p2 | 16823 | 16527 | - | 98 | MULTISPECIES: hypothetical protein |
| p2 | 17645 | 18232 | + | 195 | hypothetical protein |
| p2 | 18360 | 18238 | - | 40 | hypothetical protein KKPNMP14_55520 |
| p2 | 18452 | 18616 | + | 54 | MULTISPECIES: hypothetical protein |
| p2 | 19169 | 19453 | + | 94 | MULTISPECIES: hypothetical protein |
| p2 | 19827 | 19474 | - | 117 | MULTISPECIES: TaxD |
| p2 | 21480 | 20467 | - | 337 | MULTISPECIES: replication protein RepB |
| p2 | 22833 | 23348 | + | 171 | MULTISPECIES: molecular chaperone DnaJ |
| p2 | 23407 | 23643 | + | 78 | MULTISPECIES: hypothetical protein |
| p2 | 24320 | 24532 | + | 70 | MULTISPECIES: hypothetical protein |
| p2 | 24522 | 24764 | + | 80 | MULTISPECIES: hypothetical protein |
| p2 | 24858 | 25196 | + | 112 | MULTISPECIES: hypothetical protein |
| p2 | 25271 | 25435 | + | 54 | hypothetical protein |
| p2 | 25812 | 25570 | - | 80 | hypothetical protein BN18_3583 |
| p2 | 26135 | 26575 | + | 146 | hypothetical protein AE98_05367, partial |
| p2 | 26578 | 27738 | + | 386 | MULTISPECIES: spore coat protein CotH |
| p2 | 28466 | 28600 | + | 44 | MULTISPECIES: transcriptional regulator |
| p2 | 28551 | 28787 | + | 78 | MULTISPECIES: hypothetical protein |
| p2 | 29461 | 29751 | + | 96 | MULTISPECIES: PilX2 |
| p2 | 29776 | 32529 | + | 917 | MULTISPECIES: type IV secretion/conjugal transfer ATPase PilX3-PilX4 |
| p2 | 32539 | 33309 | + | 256 | MULTISPECIES: pilus assembly protein |
| p2 | 33588 | 34643 | + | 351 | MULTISPECIES: pilus assembly protein |
| p2 | 34838 | 35566 | + | 242 | MULTISPECIES: pilus assembly protein |
| p2 | 35572 | 36501 | + | 309 | P-type conjugative transfer protein VirB9 |
| p2 | 36498 | 37712 | + | 404 | MULTISPECIES: conjugation transfer protein TrbI |
| p2 | 37714 | 37911 | + | 65 | MULTISPECIES: hypothetical protein |
| p2 | 37908 | 38942 | + | 344 | MULTISPECIES: ATPase |
| p2 | 39185 | 40780 | + | 531 | VirD4 |
| p2 | 41410 | 41571 | + | 53 | MULTISPECIES: hypothetical protein |
| p2 | 42557 | 43819 | + | 420 | MULTISPECIES: ATPase |
| p2 | 43823 | 46150 | + | 775 | MULTISPECIES: DNA topoisomerase III |
