# Supplementary material for: Virulence and Genomic Feature of a Virulent Klebsiella pneumoniae Sequence Type 14 Strain of Serotype K2 Harboring blaNDM–5 in China
Source: Front Microbiol. 2017 Mar 23;8:335. doi: 10.3389/fmicb.2017.00335 (PMC5362587; doi:10.3389/fmicb.2017.00335)
Supplement: Supplementary file 2 [file Table_2.DOC]

Table S2. MICs for the NDM-5-carrying *Klebsiella pneumoniae* clinical isolate, transconjugant and transformant

| Antibiotic | MIC (mg/L)/antimicrobial susceptibility | | | | | |
| --- | --- | --- | --- | --- | --- | --- |
|  | *K.pneumoniae* NUHL24835 | *E. coli* transformant  NUHL24835-NDM-TOP10 | *E. coli* transconjugant  NUHL24835-NDM-J53 | *E. coli*  TOP10 | *E. coli*  J53 | |
| Penicillins | | | | | | |
| ampicillin/sulbactam | ≥32/R | ≥32/R | ≥32/R | ≤4/S | | ≤4/S |
| Monobactams | | | | | | |
| aztreonam | ≥64/R | ≥64/R | ≥64/R | ≤1/S | | ≤1/S |
| Cephalosporins | | | | | | |
| cefazolin | ≥64/R | ≥64/R | ≥64/R | ≤4/S | | ≤4/S |
| cefotetan | ≥64/R | ≥64/R | ≥64/R | ≤4/S | | ≤4/S |
| ceftriaxone | ≥64/R | ≥64/R | ≥64/R | ≤1/S | | ≤1/S |
| ceftazidime | ≥64/R | ≥64/R | ≥64/R | ≤1/S | | ≤1/S |
| Carbapenems | | | | | | |
| imipenem | ≥16/R | 8R | ≥16/R | ≤1/S | | ≤1/S |
| meropenem | ≥16/R | ≥16/R | ≥16/R | ≤0.25/S | | ≤0.25/S |
| Fluoroquinolones | | | | | | |
| ciprofloxacin | ≥4/R | 4R | 4R | ≤0.25/S | | ≤0.25/S |
| levofloxacin | ≥8/R | 4R | 4R | ≤0.25/S | | ≤0.25/S |
| Aminoglycosides | | | | | | |
| amikacin | 16/S | ≤2/S | ≤2/S | ≤2/S | | ≤2/S |
| gentamicin | ≤1/S | ≤1/S | ≤1/S | ≤1/S | | ≤1/S |
| tobramycin | 8/I | ≤1/S | ≤1/S | ≤1/S | | ≤1/S |
| Others | | | | | | |
| trimethoprim/sulfamethoxazole | ≥320/R | ≤20/S | ≤20/S | ≤20/S | | ≤20/S |
| tigecycline | <0.5S | <0.5S | <0.5S | <0.5S | | <0.5S |
