# Supplementary figures and images for: Virulence and Genomic Feature of a Virulent Klebsiella pneumoniae Sequence Type 14 Strain of Serotype K2 Harboring blaNDM–5 in China
Source: Front Microbiol. 2017 Mar 23;8:335. doi: 10.3389/fmicb.2017.00335 (PMC5362587; doi:10.3389/fmicb.2017.00335)

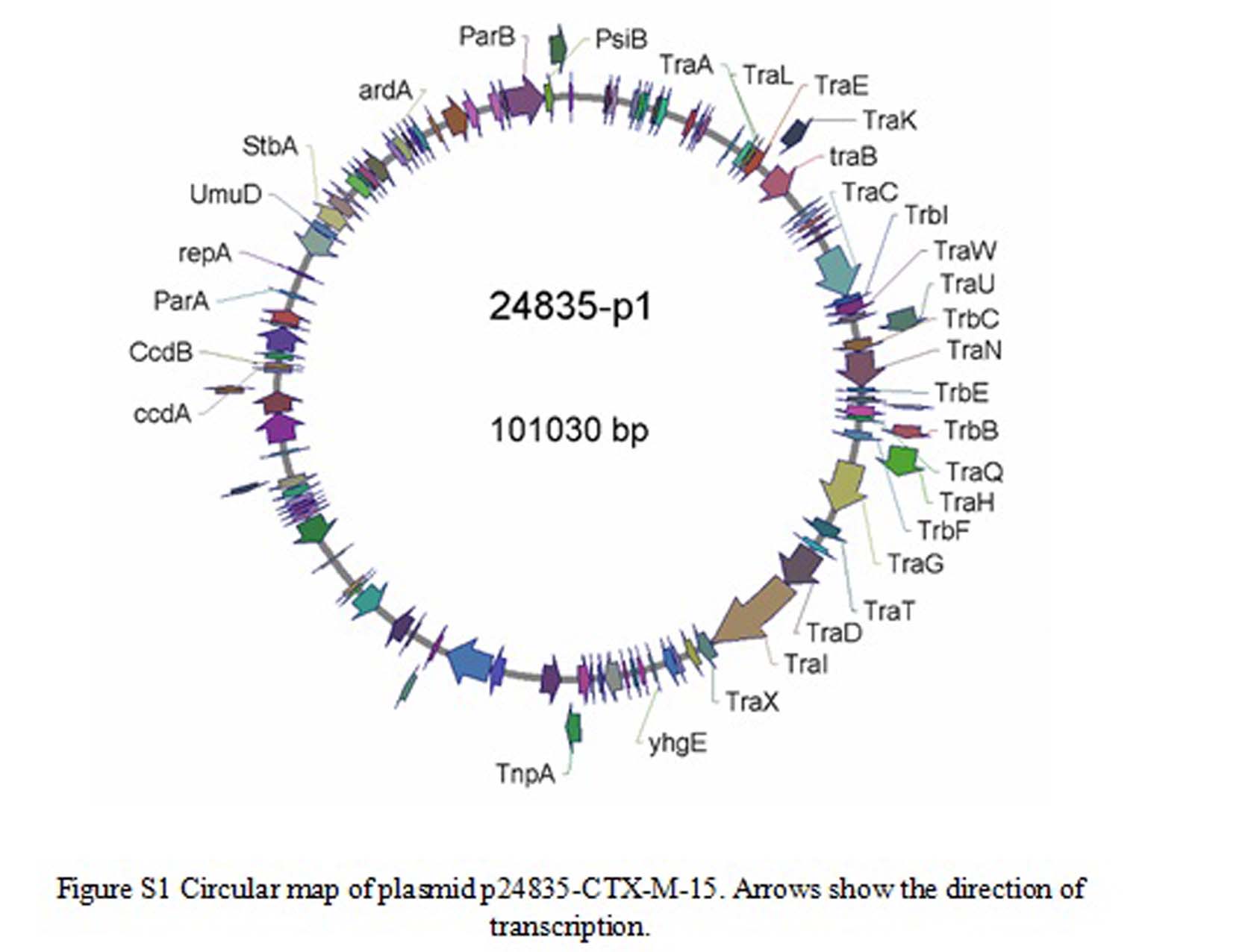

Supplement: Supplementary file 3 [file Image_1.JPEG]

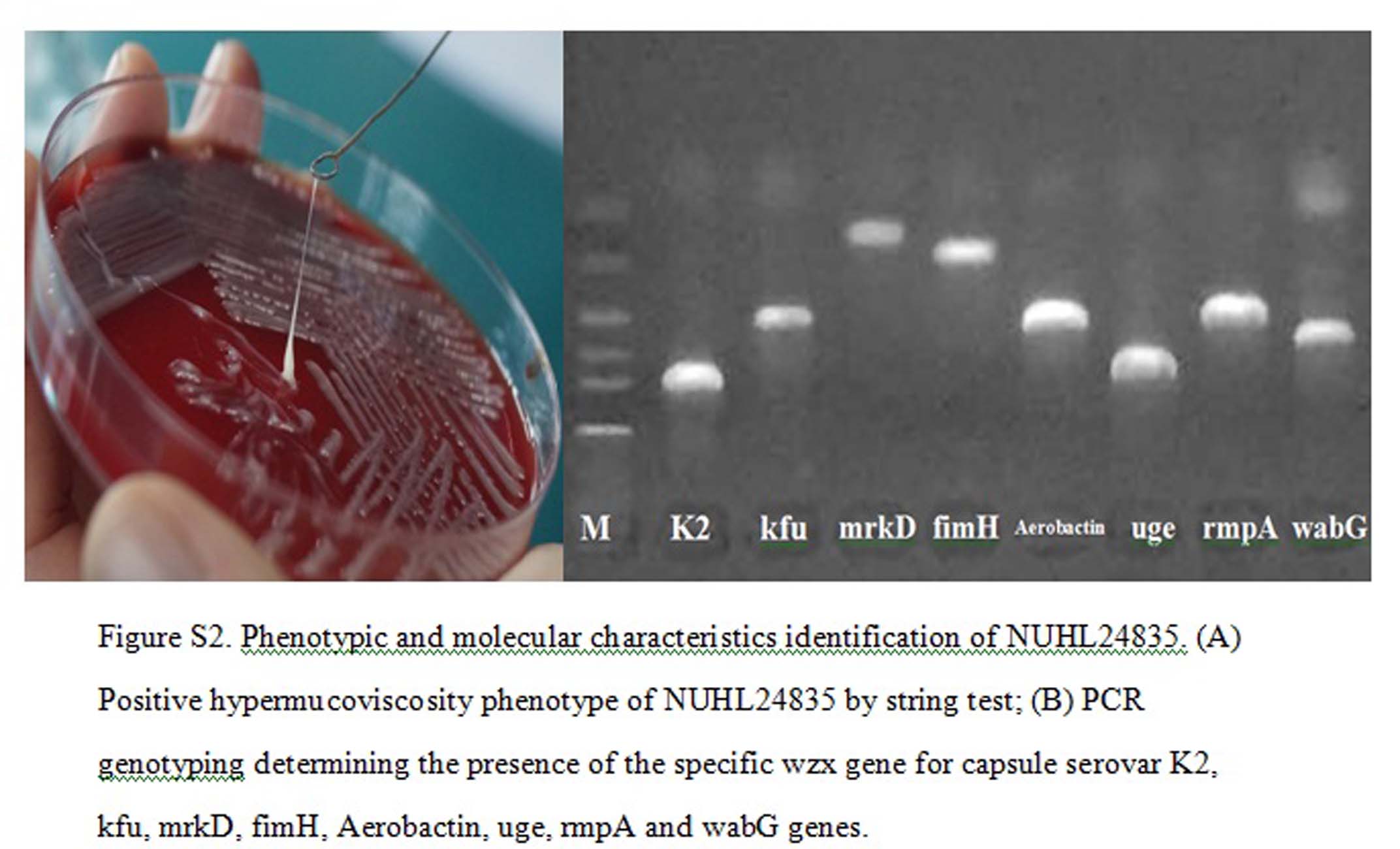

Supplement: Supplementary file 4 [file Image_2.JPEG]
